# 504 Starting a Pediatric Burn Center: Challenges Faced in an Underserved Patient Population

**DOI:** 10.1093/jbcr/irac012.135

**Published:** 2022-03-23

**Authors:** Nicole M Kopari, Jessica A Zagory, Kristen Lindsey, Herb A Phelan, Jeffrey E Carter

**Affiliations:** Children's Hospital New Orleans, New Orleans, Louisiana; Louisiana State University Health Sciences Center New Orleans, New Orleans, Louisiana; Children's Hospital New Orleans, New Orleans, Louisiana; LSUHSC-New Orleans Department of Surgery, New Orleans, Louisiana; University Medical Center- New Orleans, New Orleans, Louisiana

## Abstract

**Introduction:**

Burn injury is the third most common cause of childhood injury resulting in death. The CDC recognizing the South as having the highest rate of pediatric burn deaths in the U.S. Unfortunately, 10% of all child abuse cases involve burn injuries and 20% of all pediatric burn admissions are due to nonaccidental trauma. Our study demonstrated that aftercare was a major challenge in starting a pediatric burn center. We analyzed the rate of lost to follow-up in burn-injured children following surgery and our steps to address this need in our community through key partnerships within our state.

**Methods:**

Our study is a single center review of pediatric burn-injured children undergoing surgery from 01/01/2021 through 09/30/2021. Lost to follow-up was defined as three or more consecutive months without clinic or telemedicine visits despite three of more documented communication attempts by attending surgeons and/or clinic staff. Children requiring child protective services (CPS) for suspected nonaccidental trauma were compared to those where nonaccidental trauma was not suspected. All children sustained burn injuries of sufficient severity to require excision and autograft with follow-up in the outpatient clinic. Families were provided with an after-visit summary reviewing the clinic appointment, transportation and meal assistance, and they received a call prior to clinic to remind them of the scheduled appointment.

**Results:**

A total of 35 children required surgery with outpatient follow-up per protocol. 23% of the patients required CPS investigations. We reviewed 151 subsequent clinic visits and the associated cancellations, rescheduled appointments, and no-show visits. Children under the care of CPS had a higher rate of being lost to follow-up (50%) compared to other children (17%). Parents undergoing CPS investigation were 4x less likely to provide cancellation notice. Children placed in foster care had no cancellations, reschedules appointments, or missed visits despite a higher number of clinic visits overall.

**Conclusions:**

Children suffering nonaccidental injuries represent an exceptionally vulnerable portion of our population. Burn injuries often are a public and personal reminder of severe trauma. CPS works to find a balance in securing a safe home while attempting to maintain a family unit. Our work demonstrated an unacceptably high rate of loss to follow-up for children requiring surgical intervention after injury especially in those with concerns for nonaccidental etiologies. As a result, our burn surgeons led an initiative with statewide burn directors and our state’s emergency response network to engage the state’s CPS department. Our goal was to raise awareness and increase education for CPS social workers and foster families on burn injury and aftercare needs.

